# Variation in bill surface area is associated with local climatic factors across populations of the plain laughingthrush

**DOI:** 10.1002/ece3.10535

**Published:** 2023-09-27

**Authors:** Pengfei Liu, Yingqiang Lou, Jingxiao Yao, Linghui Wang, Anders Pape Møller, Yuehua Sun

**Affiliations:** ^1^ School of Life Science and Technology Longdong University Qingyang China; ^2^ Key Laboratory of Animal Ecology and Conservation Biology Institute of Zoology, Chinese Academy of Science Beijing China; ^3^ Ecologie Systématique Evolution, Université Paris–Sud, CNRS, AgroParisTech Université Paris–Saclay Orsay Cedex France

**Keywords:** Allen's rule, bill morphology, *Garrulax davidi*, heat dissipation, tarsus length

## Abstract

Recent studies have found that avian bill and tarsus morphology may have evolved in response to climatic conditions, and these organs play important roles in thermoregulation and water retention in extreme environments. Here, we examined whether bill surface area and tarsus length were associated with climatic conditions in the plain laughingthrush, *Garrulax davidi*, which mainly occurs in north China and occupies several climatic zones from east to west. We measured bill surface area and tarsus length in 321 adults from 11 populations, almost encompassing all habitat types of the species. We analyzed the relationships among these morphological traits and local climatic factors. Bill surface area was positively correlated with maximum temperature, indicating that bill surface area tended to be larger in hotter environments. Furthermore, we found a negative relationship among bill surface area and winter precipitation, indicating that bill surface area tended to be larger in arid areas. However, we did not find any relationships between tarsus length and climatic factors. These results suggest that local climates may shape the evolution of bill morphology divergence, and summer seems to be the critical season for thermoregulation in this temperate zone passerine.

## Graphical abstract

Our study first provided evidence for Allen's rule that bill size was associated with environmental condition in the plain laughingthrush. This study adds the evidence of evolution of bill and tarsus morphology in birds.

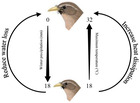

## 1 INTRODUCTION

Allen's rule proposed that the projecting parts of the body of endotherms is longer in tropical areas facilitating dissipation of excess heat, while it is shorter for the retention of heat in cold regions (Allen, 1877; Campbell‐Tennant et al., 2015; Friedman et al., 2017; Miller et al., 2018; Nudds & Oswald, 2007). This rule suggested that in cold climates, heat loss of homeotherms could be reduced through decreasing surface area relative to volume of a body and by reducing the length of body appendages (Allen, 1877).

The avian bill is a multifunctional organ, and its morphology is closely linked with foraging ecology (Grant & Grant, 2006; Probst et al., 2022). Recent studies have documented that bill size was modified by environmental temperature, such as minimum temperature in winter (Danner et al., 2015), and maximum temperature in summer (Greenberg, Cadena, et al., 2012; Greenberg, Danner, et al., 2012), both across and within single species (Danner et al., 2015; Greenberg, Cadena, et al., 2012; Greenberg, Danner, et al., 2012). The bill plays important roles in thermoregulation through heat exchange with environment and non‐evaporative cooling (Greenberg, Cadena, et al., 2012; Greenberg & Danner, 2012; Greenberg, Danner, et al., 2012; Probst et al., 2022; Tattersall et al., 2017). For example, up to 60% of the body heat production could dissipate through the bill in the toco toucan, *Ramphastos toco* (Tattersall et al., 2009). Excess heat could dissipate through bill surface to reduce heat load and avoid hyperthermia with no water loss; in other words, heat dissipated from the bill is dry heat (Probst et al., 2022). Therefore, species or populations living in hot and arid environments should have larger bill size, and non‐evaporative cooling aid in water retention (Greenberg & Danner, 2012; Probst et al., 2022; Scott et al., 2008). However, different results were also found in other species, for example, in LeConte's thrasher, *Toxostoma lecontei*, which occupies the hottest and most arid climates within its genus, the researchers found a negative correlation between bill size and maximum temperature of the hottest month significantly (Probst et al., 2022). In hot and arid environments, larger bills were thought to dissipate more heat and reduce water loss. While in some extreme environments in which fresh water was restricted, birds need to increase heat loss through non‐evaporative cooling and convection when the ambient temperature surpasses body temperature, such as panting and spread their wings (Greenberg & Danner, 2012; McKechnie et al., 2021; Probst et al., 2022; Scott et al., 2008). In this situation, the bill functions as an organ of heat absorbtion. Bill morphology was also modified by environmental temperature and humidity. Gerson et al. (2014) found that higher relative humidity would decrease the evaporative cooling function in desert birds. Therefore, to figure out the relationships between bill morphology and climatic factors, more studies are needed.

The tarsus is featherless in most species and exposed body areas, which is related to heat loss in birds (Ederström & Brumleve, 1964; Johansen & Wesley Millard, 1973; Nudds & Oswald, 2007; Steen & Steen, 1965). In the pigeon, *Columba livia*, legs and feet play major roles in whole‐body thermoregulation, both in rest and flight (Martinrau & Larochelle, 1988). The longer legged birds living in colder environment experience greater metabolic costs, as the longer legs elevate the body into a zone with higher wind speeds, which caused increasing convective cooling (Cartar & Morrison, 2005). Based on the studies of shorebirds breeding in the arctic, Cartar and Morrison (2005) argued that body‐supporting appendages, especially legs of homeotherms, may be shorter in colder climates which can reduce metabolic cost. Other studies found correlations between environmental temperatures and appendage correlations with minimum temperature of occupied habitats in birds (Alhajeri et al., 2020; Friedman et al., 2017; Greenberg, Cadena, et al., 2012; Greenberg, Danner, et al., 2012), which are consistent with Allen's rule.

**FIGURE 1:**
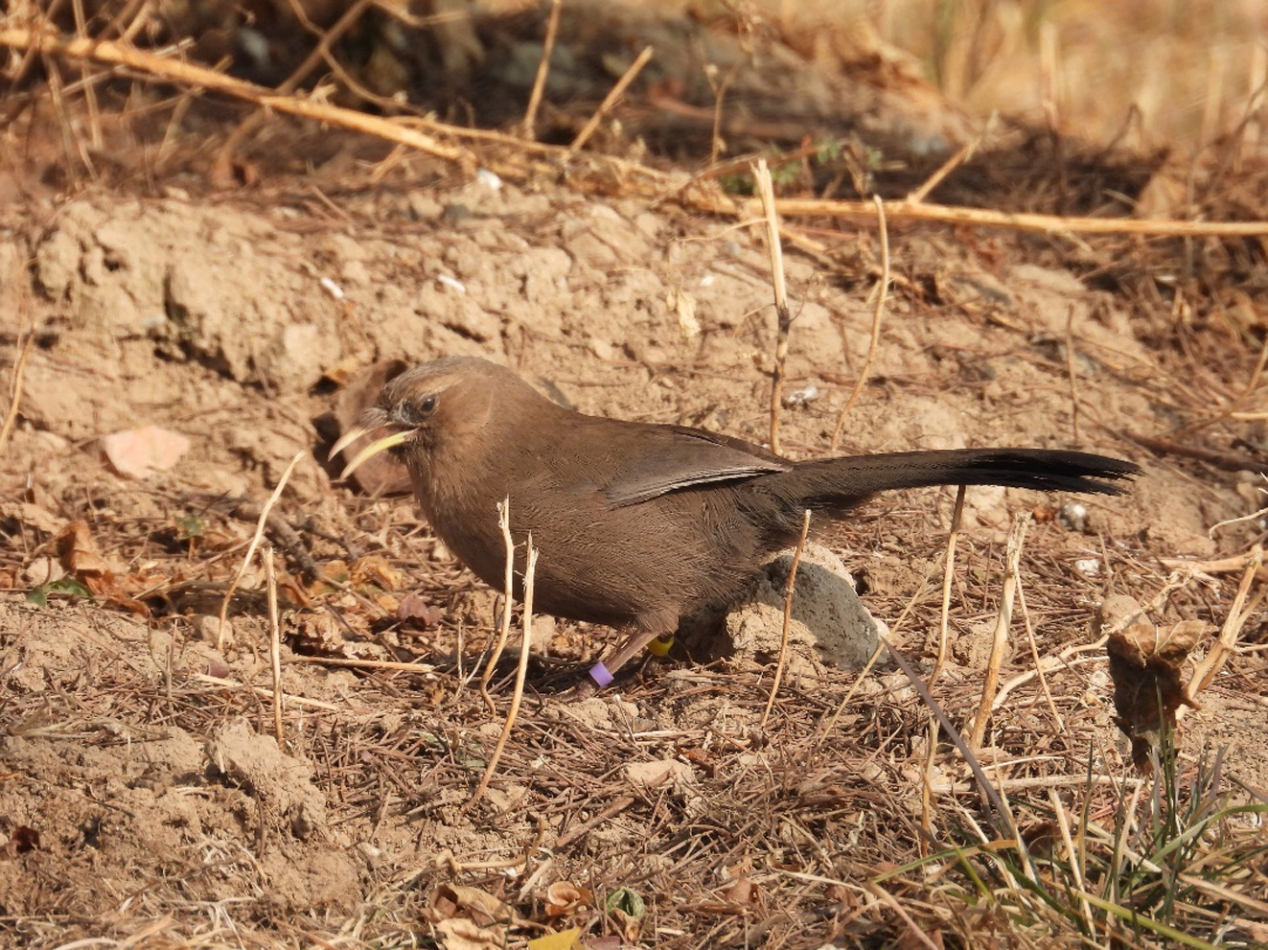
Picture of plain laughingthrush (*Garrulax davidi*).

To better understand the evolution of bill and tarsus morphology in birds, we selected the plain laughingthrush (*Garrulax davidi*) as our research subject (Figure 1), which distributed in temperate zones and is a Chinese endemic (Liu & Sun, 2018). Plain laughingthrush is a suitable species to investigate the effects of climate on the evolution of bill and tarsus morphology for the following reasons: first, it is abundant and widely distributed in North China, which across most of temperate eco‐climatic zones, and these regions are significantly differed in climates. Second, it is a resident species and experiences hot summer and cold winter of temperate zones. In this study, we aimed to investigate (1) whether there are relationships between bill surface area and climatic factors; (2) whether tarsus length was related with local climatic factors. According to Allen's rule, we predicted that the bill surface area tends to be larger in hotter and more arid habitat populations, and tarsus length tends to be shorter in colder habitats populations.

## 2 MATERIALS AND METHODS

### 2.1 Bird capture and measurement

**FIGURE 2:**
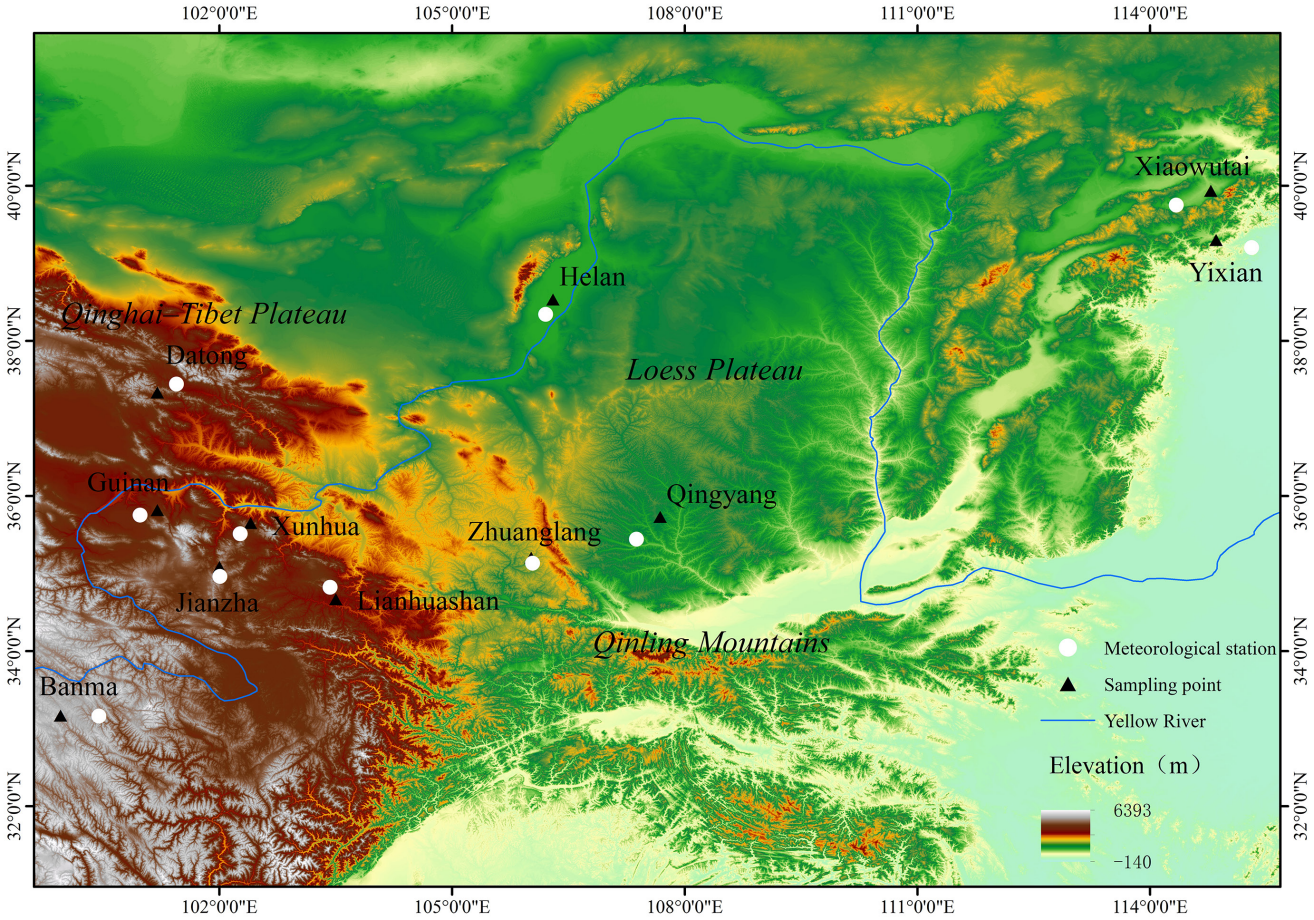
The geographic locations of 11 sampling sites in this study, including three field sampling sites (Lianhuashan, Zhuanglang, and Qingyang) and museum specimen from eight sites (Datong, Guinan, Banma, Jianzha, Xunhua, Helan Mountain, Xiaowutai, and Yixian). The solid black points represent the sampling sites.

We collected morphological data on 321 adult plain laughingthrushes from 11 geographic populations (Figure 2), including three field sampling sites in central China, which are Lianhuashan National Natural Reserve, the eastern edge of Qinghai–Tibet Plateau, southwest of Gansu Province; Zhuanglang County at western Liupanshan Mountains, Gansu Province; the Campus of Longdong University and its surrounding areas at Qingyang city, in the interior of Loess Plateau, eastern of Gansu province. In total, we captured 257 adult plain laughingthrushes in these three sites (Lianhuashan, in 2015, Zhuanglang and Qingyang in 2019–2021) with walk‐in traps and mist‐nets from October to December. We also measured 64 specimens which are deposited in National Animal Collection Resource Center, Institute of Zoology, & The Chinese Academy of Sciences (see Table S1 for the detail information of collecting sites and number of individuals).

These sampling sites encompass most geographic and climatic gradients within the natural distributions of plain laughingthrush in China (Zheng, 2017). For each bird collected in the field and stored specimen, we measured the length, width and height of the bill, right wing length (carpal joint to the tip of the longest primary feather unflattened), and right tarsus length (joint of tibiotarsus and tarsometatarsus to the distal edge of the last undivided scute on the anterior surface of the leg) with digital calipers (all linear distance nearest to 0.01 mm). For capture birds, sex is identified by the differences in body measurements (Liu & Sun, 2018) and the differences in singing behavior (Liu et al., 2022). For the specimens, we recorded the collection site and sex from the specimen tags. Bill surface area was calculated by the formula: bill surface area = (bill width + bill depth)/4 × bill length × *π* (Danner & Greenberg, 2012).

### 2.2 Climate data collection

We selected the meteorological stations which located nearest to each sampling site in this study in order to obtain local weather information and extract climatic variables (Figure 2). The climatic data of those meteorological stations were downloaded from the website of China Meteorological Administration (https://cma.gov.cn) for nearly 20 years (from the year 2000 to 2018). We extracted four climatic variables from obtained meteorological data for each sampling site: maximum temperature for the hottest month, minimum temperature for the coldest month, mean summer precipitation, and mean winter precipitation, and then we calculated the average climatic variables for further analyses.

### 2.3 Statistical analysis

We investigated whether the bill surface area and tarsus length varied among sampling sites, two Kruskal–Wallis tests were used. A linear mixed model (LMM) was used to investigate whether bill surface area was predicted by climate variables. The minimum and maximum temperature, precipitation in winter and summer, collection year and sex were treated as fixed variables. The bill surface area was log transformed to meet normal distribution before analyzing. A LMM was used to investigate whether tarsus length was predicted by climate variables. The model included minimum and maximum temperature, winter and summer precipitation, collection year, and sex. Variance Inflation Factor (VIF) was used to check the collinearity of these factors and ensure the VIF values <2.5 (Johnston et al., 2018). The final models included both minimum and maximum temperatures, winter and summer precipitation and sex. Collection area was used as a random effect in both models. The significance level of the predictors was tested using the function Anova in the R package “car” (Fox et al., 2012). All LMMs were fitted with the *lmer* function from the *lme4* package (Bates et al., 2014) and all statistical analyses were conducted in R (R Core Team, 2015).

## 3 RESULTS

**FIGURE 3:**
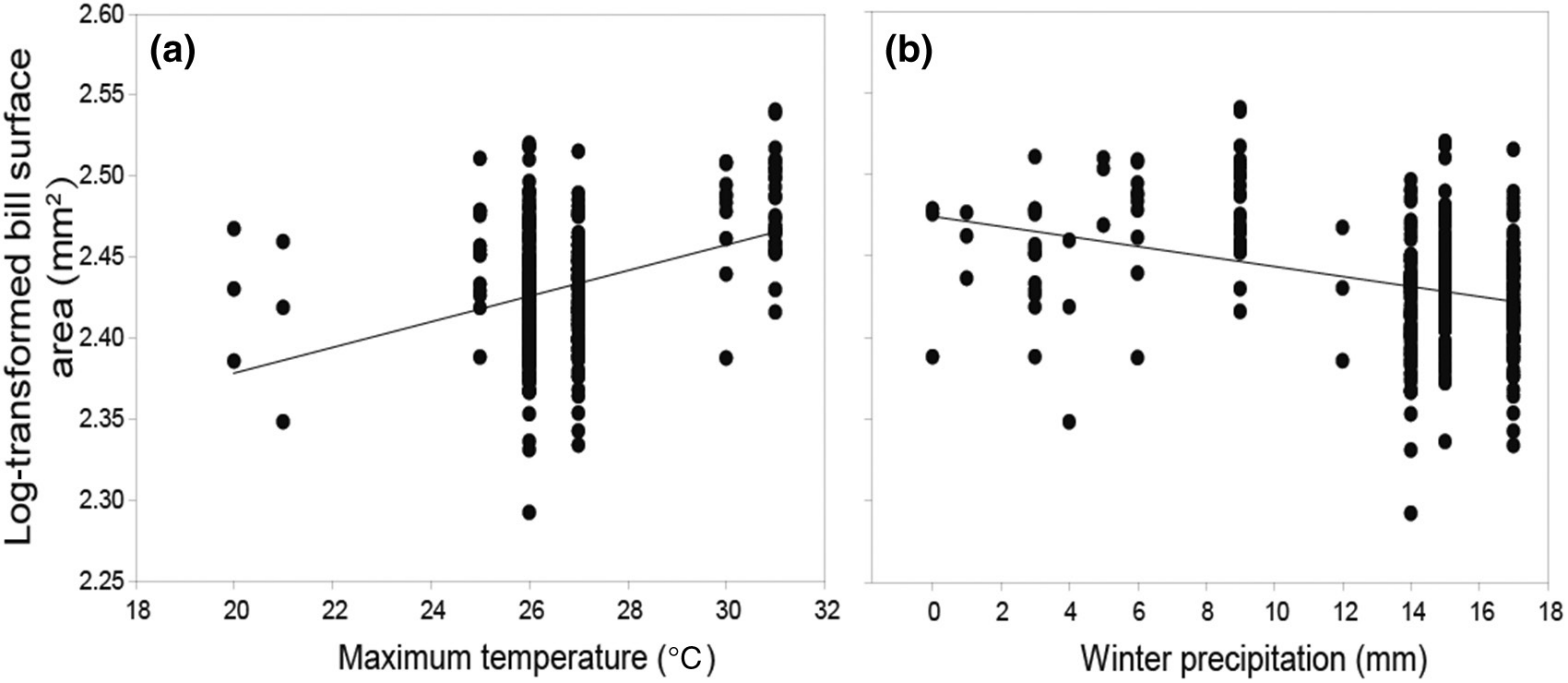
Relationship between climatic factors and bill surface area in plain laughingthrush: (a) maximum temperature for the hottest month (mean maximum temperature for July) and (b) winter precipitation (mean winter precipitation). Each point represents an individual.

Bill surface area varied among populations (*χ* ^2^ = 74.091, *p* < .001, Table 1), with Guinan population having the smallest bill surface area (mean ± SD = 257.59 ± 32.74 mm^2^), and the Helan Mountain population has the largest bill surface area (mean ± SD = 311.99 ± 15.64 mm^2^). The bill surface area was larger in males than in females (Table 2). Furthermore, the bill surface area was positively correlated with maximum temperature (Table 2), indicating bill surface area was larger in hotter habitats (Figure 3a). Bill surface area was negatively correlated with winter precipitation (Table 2), indicating that bill surface area was larger in habitats with lower winter precipitation (Figure 3b).

**TABLE 1:** Summary of bill size and tarsus length in 11 collection locations.

	Locations	Range (mm^3^)	Mean ± SE
Bill size (mm)	Qingyang	216.72–331.00	264.65 ± 20.14
	Zhuanglang	195.86–313.48	262.73 ± 20.36
	Lianhuashan	216.72–331.00	272.90 ± 22.73
	Xiaowutai	243.93–322.42	298.32 ± 23.82
	Yixian	260.37–346.94	301.31 ± 21.20
	Helan	294.14–323.33	311.99 ± 15.64
	Datong	244.24–323.95	282.45 ± 20.66
	Xunhua	244.24–300.93	281.34 ± 32.14
	Jianzha	272.88–299.44	287.35 ± 13.44
	Guinan	222.82–287.84	257.59 ± 32.74
	Banma	242.90–293.15	268.40 ± 25.14
Tarsus length (mm)	Qingyang	29.43–36.10	31.77 ± 1.18
	Zhuanglang	27.24–33.99	30.87 ± 1.49
	Lianhuashan	26.80–33.49	30.18 ± 1.47
	Xiaowutai	29.57–32.30	31.14 ± 0.84
	Yixian	29.55–33.65	31.99 ± 1.06
	Helan	29.50–33.30	31.93 ± 2.11
	Datong	28.26–33.79	31.88 ± 1.48
	Xunhua	28.26–32.69	30.48 ± 2.22
	Jianzha	29.35–34.31	32.06 ± 2.51
	Guinan	31.18–31.98	31.62 ± 0.41
	Banma	31.81–32.41	32.08 ± 0.31

**TABLE 2:** Parameter estimates of factors relating to bill size in plain laughingthrush by linear mixed model.

Predictors	Estimate	SE	*χ* ^2^	*p*
Winter precipitation	−0.006	0.002	13.463	**<.001**
Summer precipitation	−0.001	0.001	0.014	.905
Maximum temperature	0.013	0.003	14.845	**<.001**
Minimum temperature	−0.005	0.003	2.093	.148
Sex	−0.074	0.008	77.479	**<.001**

*Note*: The significant factors were shown in bold.

Tarsus length varied across populations (*χ* ^2^ = 120.510, *p* < .001, Table 1), with the Banma population having the shortest tarsi (mean ± SD = 268.40 ± 25.14 mm), Xunhua population having the longest tarsi (mean ± SD = 281.34 ± 32.14 mm). We found that the tarsus length of males was larger than that of females (Table 3), and the climate variables were not associated with tarsus length (Table 3).

**TABLE 3:** Relationships of tarsus length with climatic factors in plain laughingthrush by linear mixed model.

Predictors	Estimate	SE	*χ* ^2^	*p*
Minimum temperature	0.032	0.106	0.094	.760
Maximum temperature	−0.027	0.095	0.082	.774
Summer precipitation	0.002	0.004	0.266	.606
Winter precipitation	−0.080	0.059	1.828	.176
Sex	−0.767	0.135	32.198	**<.001**

*Note*: The significant factors are shown in bold.

## 4 DISCUSSION

Our results showed that the bill surface area and tarsus length of male plain laughingthrushes were larger than those of females, which is in line with Liu and Sun (2018). Furthermore, our results support our predictions that populations in hotter habitats have larger bills than those in colder habitats. The bill surface area was also negatively associated with winter precipitation. However, there was little support for the relationship between tarsus length and climatic factors. Our study highlights the multiple variables in explaining the evolution of bill surface area in plain laughingthrush.

Populations with higher summer temperatures have larger bills in the plain laughingthrush, a result consistent with Allen's rule (Allen, 1877; Greenberg, Cadena, et al., 2012; Greenberg, Danner, et al., 2012; McQueen et al., 2022). The plain laughingthrush is widely distributed in north China and adapts to temperate climates, optimal temperatures of this species are broad, it is not good at resisting high temperatures, but it deals well with low temperatures (Liu et al., 2023). The ambient temperature the species experienced is lower than its body temperature even in the hottest month, and most occupied habitats of the species were aridity and fresh water was restricted (Liu et al., 2023), with a larger bill being advantageous in excess heat dissipation and water retention. Therefore, larger bills may evolve to adapt the high temperature in summer, especially in species which occupying arid habitats (Greenberg, Danner, et al., 2012; Luther & Greenberg, 2014). Furthermore, for animals living in seasonal climates, the morphological traits may be linked with thermoregulation evolved through the trade‐off between the need for heat dissipation in summer and heat conservation in winter (Fan et al., 2019).

We did not find that tarsus length was associated with climatic factors in our birds, and this result was not in line with Allen's rule (Miller et al., 2018; Salewski & Watt, 2017). Heat loss in birds occurs primarily from unfeathered areas, including the bill, legs, face, and feet (Phillips & Sanborn, 1994; Symonds & Tattersall, 2010; VanderWerf, 2012). Small unfeathered areas could reduce the relative radiating surface area to the body, which can reduce the metabolic costs in convective cooling (Cartar & Morrison, 2005). In addition, the bill can tuck under feathers and play an important role in thermoregulation in birds, and this thermoregulatory behavior was utilized more by species with larger bills (Ryeland et al., 2017), and the tarsus can be covered with feathers during resting behavior (sitting and standing on one leg), which could regulate the area of the body surface exposed to reduce heat loss, a study had found that the bird species with relatively short legs were less reliant on thermoregulatory behavior of stand on one leg to minimize heat loss from these bare appendages (Ryeland et al., 2019). Therefore, the importance of thermoregulation of bill and tarsus is differed among different populations and species, and experienced different selective pressures by ecological factors.

Bill surface area was negatively correlated with the level of winter precipitation, and this result was not in line with other species (Greenberg, Cadena, et al., 2012; Probst et al., 2022). In winter, the plain laughingthrush was resident and they may face water restrictions in some habitats, such as little precipitation and the water resources are frozen. They could only obtain water from food resources and body metabolism in this situation, how to reduce water loss may be an essential selective pressure. A recent study found that bill size increase (7.37%) could be reduced by 16.2% water loss in California Savannah sparrow (*Passerculus sandwichensis*) populations (Benham & Bowie, 2021). Therefore, the functions of bill size in reducing water loss may drive the morphological differentiation in plain laughingthrush.

In summary, our study first provided evidence for Allen's rule that bill surface area was associated with environmental condition in the plain laughingthrush. To fully understand the evolution of bill and tarsus morphology in birds, multiple environmental factors are needed to be considered in future research.

## AUTHOR CONTRIBUTIONS

**Pengfei Liu:** Conceptualization (equal); data curation (equal); formal analysis (equal); methodology (equal). **Yingqiang Lou:** Conceptualization (equal); formal analysis (equal). **Jingxiao Yao:** Data curation (equal). **Linghui Wang:** Data curation (equal). **Anders Pape Møller:** Writing – original draft (equal). **Yuehua Sun:** Conceptualization (equal); funding acquisition (equal); methodology (equal); project administration (equal); writing – original draft (equal).

## CONFLICT OF INTEREST STATEMENT

The authors declare no conflicts of interest.

## Supporting information


Table S1

## Data Availability

Data are available in the supplementary materials.
