# Supplementary material for: Variation in bill surface area is associated with local climatic factors across populations of the plain laughingthrush
Source: Ecol Evol. 2023 Sep 27;13(9):e10535. doi: 10.1002/ece3.10535 (PMC10534077; doi:10.1002/ece3.10535)
Supplement: Supplementary file 1 — Table S1 [file ECE3-13-e10535-s001.zip › ece310535-sup-0001-TableS1.docx]

Supplemental table 1. The information of Plain Laughingthrush data from 11 geographical populations

| Location | Province | Sample size | Condition |
| --- | --- | --- | --- |
| Qingyang | Gansu | 85 | Field sampling |
| Zhuanglang | Gansu | 96 | Field sampling |
| Lianhuashan | Gansu | 76 | Field sampling |
| Xiaowutai | Hebei | 10 | Species from museum |
| Yixian | Hebei | 26 | Species from museum |
| Helan | Ningxia | 3 | Species from museum |
| Datong | Qinghai | 13 | Species from museum |
| Xunhua | Qinghai | 3 | Species from museum |
| Jianzha | Qinghai | 3 | Species from museum |
| Guinan | Qinghai | 3 | Species from museum |
| Banma | Qinghai | 3 | Species from museum |
